# Differences in the direct effects of various type 2 cytokines on functions of blood eosinophils from healthy subjects

**DOI:** 10.5415/apallergy.0000000000000157

**Published:** 2024-08-16

**Authors:** Yutaka Ueda, Kazuyuki Nakagome, Kazuki Katayama, Hidetoshi Iemura, Sachiko Miyauchi, Toru Noguchi, Takehito Kobayashi, Tomoyuki Soma, Toshiko Itazawa, Makoto Nagata

**Affiliations:** 1 Department of Respiratory Medicine, Saitama Medical University, Saitama, Japan; 2 Department of Pediatrics, Saitama Medical University, Saitama, Japan; 3 Allergy Center, Saitama Medical University, Saitama, Japan

**Keywords:** Eosinophils, interleukin-4, interleukin-13, thymic stromal lymphopoietin

## Abstract

**Background:**

Eosinophil inflammation often persists in the airways of severe asthmatics, even under treatment with high-dose inhaled corticosteroids. Biologics for various type 2 cytokines have been recently developed for corticosteroid-resistant, eosinophil-dominant, severe asthma. However, it is unclear whether these biologics act directly on eosinophils.

**Objective:**

In this study, we examined whether various type 2 cytokines targeted by biologics can directly modify the functions of eosinophils obtained from the peripheral blood of healthy individuals.

**Methods:**

Peripheral eosinophils of healthy subjects were purified by conventional negative-depletion methods using anti-CD16 beads to avoid the priming effect (i.e., stimulation *in vitro*) to the maximum extent possible. Eosinophils were stimulated with interleukin (IL)-4, IL-5, IL-13, or thymic stromal lymphopoietin (TSLP), and eosinophil adhesiveness to recombinant human-intercellular adhesion molecule (ICAM)-1 was evaluated by eosinophil peroxidase assays. The effect of these cytokines on eosinophil superoxide anion (O_2_^−^) generation was evaluated by the superoxide dismutase-inhibitable reduction of cytochrome C. To determine whether eosinophil degranulation was induced, the concentration of eosinophil-derived neurotoxin (EDN) in the supernatant was measured using enzyme-linked immuno sorbent assay.

**Results:**

As reported previously, at 100 pM, IL-5 increased eosinophil adhesiveness to ICAM-1, O_2_^−^ generation, and EDN release. Conversely, at concentrations up to 10 nM, IL-4, IL-13, and TSLP did not induce eosinophil adhesiveness, O_2_^−^ generation, or EDN release.

**Conclusion:**

Type 2 cytokines other than IL-5 do not directly affect the functions of eosinophils from healthy individuals when used at clinical concentrations. These findings suggest that eosinophils play little, or no, direct role in the effects of anti-IL-4 receptor α or anti-TSLP antibody on severe asthma.

## 1. Introduction

Bronchial asthma is characterized by eosinophilic airway inflammation, airway hyperresponsiveness (AHR), and reversible airway obstruction [1]. Various cell types contribute to the pathophysiology of airway inflammation in asthma. Of these, eosinophils play an important role in the pathogenesis of asthma, primarily through the release of inflammatory mediators and specific granule proteins such as eosinophil-derived neurotoxin (EDN) [2, 3].

Type 2 cytokines, such as interleukin (IL)-4, IL-5, and IL-13, which are mainly produced by T helper 2 (Th2) lymphocytes or group 2 innate lymphoid cells (ILC2), are also involved in the development of bronchial asthma [4]. For example, IL-4 plays a role in the activation of B cells, B cell differentiation into plasma cells, and the production of immunoglobulin (Ig) E, which is involved in allergic responses by enhancing cross-linking of allergens in mast cells or basophils [5]. There are 2 types of receptors (Rs) for IL-4/IL-13 (type-I IL-4R and type-II IL-4R), and IL-4 can bind to type-I and type-II IL-4Rs [5]. Type-I IL-4R is highly expressed mainly on hematopoietic cells, whereas type-II IL-4R is highly expressed on cells other than hematopoietic cells. Whether IL-4R is functionally expressed in eosinophils has not been fully clarified. One report suggests that human eosinophils express a functional IL-4 receptor [6], whereas another report suggests that type-I IL-4R is mainly expressed inside eosinophils and not at the surface [7], suggesting the limited role of IL-4 in eosinophil activation. IL-5 plays a crucial role in the differentiation of eosinophils in bone marrow, as well as eosinophil activation and survival at the site of inflammation, including airways. IL-5R is expressed in eosinophils and basophils, suggesting IL-5 can directly activate eosinophils *in vitro*. IL-13 can directly induce AHR, mucus hypersecretion, and the contraction of airway smooth muscle cells. IL-13 binding is limited to type-II IL-4R, which is highly expressed on nonhematopoietic cells including airway epithelial cells, endothelial cells, and smooth muscle cells [5]. The role of epithelial cytokines, such as IL-33 and thymic stromal lymphopoietin (TSLP), in the development of innate immune responses has recently been highlighted [8]. After damage has been caused to epithelial cells by allergens through protease activity or by viral infection, the release of IL-33 or TSLP from epithelial cells activates ILC2 to produce type 2 cytokines, including IL-5 [8]. TSLP receptor (TSLPR) is expressed in ILC2, dendritic cells (DCs), and basophils [9]. Furthermore, some reports suggest that TSLPR is also expressed on eosinophils [10] and TSLP directly activates eosinophils *in vitro* [10-12]. In general, these cytokines cooperate to induce eosinophilic inflammation in the airway of individuals with asthma through adaptive and innate immune responses.

The efficacy of biologics specific for these type 2 cytokines and/or IgE has been demonstrated in a clinical setting over the years [13]. When asthma is not controlled by standard therapy, such as high doses of inhaled corticosteroids (ICS) in conjunction with a long-acting beta-agonist supplementation, therapy with biologic agents is considered [13]. To date, omalizumab, mepolizumab, benralizumab, dupilumab, and tezepelumab have been approved for use in Japan [13]. Omalizumab, an anti-IgE antibody (Ab), suppresses allergen-induced mast cell activation and is applicable for the treatment of severe cases of asthma, urticaria, and seasonal allergic rhinitis in our country. Mepolizumab, anti-IL-5 Ab, and benralizumab, anti-IL-5R Ab, directly suppress peripheral eosinophil counts and are well suited for treating eosinophilic airway inflammation. Mepolizumab is applicable for treating eosinophil-dominant severe asthma and eosinophilic granulomatosis with polyangiitis, and benralizumab is applicable for treating severe eosinophilic asthma. Dupilumab, anti-IL-4R Ab, which suppresses the IL-4 and IL-13 signaling, is applicable for treating severe cases of asthma, chronic sinusitis with polyps, and atopic dermatitis. Although treatment with dupilumab transiently increases blood eosinophil counts, it subsequently decreases eosinophil counts. A recent report suggested that dupilumab also suppresses eosinophilic airway inflammation in asthmatics [14]. Furthermore, tezepelumab, an anti-TSLP Ab, is applicable for the treatment of severe asthma and suppresses eosinophilic airway inflammation [15]. However, the main mechanism of action for the suppression of eosinophilic inflammation by tezepelumab remains unknown, as TSLPR is expressed in ILC2, DCs, basophils, and eosinophils, as described above. Thus, although these biologic agents are effective for treating severe allergic diseases, including asthma and eosinophilic inflammation, their mechanism(s) of action, especially their direct effect on eosinophils, has not been fully clarified to date.

We therefore examined the direct effects of IL-4, IL-13, and TSLP on the functions of eosinophils obtained from the peripheral blood of healthy individuals and compared the findings to those obtained using IL-5. We found that at concentrations up to 10 nM, IL-4, IL-13, and TSLP did not activate peripheral blood eosinophils or promote adhesion to intercellular adhesion molecule (ICAM)-1, generation of superoxide anion (O_2_^−^) or degranulation. These findings indicate that anti-IL-4Rα Ab (dupilumab) or anti-TSLP Ab (tezepelumab) have little or no direct effects on eosinophils.

## 2. Materials and methods

### 2.1. *Preparation of eosinophils*

Eosinophils were obtained from the peripheral blood of nonallergic healthy individuals, defined as subjects with no history of allergic disease and no allergy-related symptoms with a peripheral blood differential eosinophil count of <5%. This study was approved by the Ethics Committee of Saitama Medical University. We obtained written informed consent from each participant of this study before blood sample collection. Eosinophils were isolated using Percoll density gradient centrifugation and negative selection by anti-CD16 Ab-coated magnetic beads (Miltenyi Biotec, Auburn, CA, USA), as described previously [16-21]. Eosinophils were purified using this conventional method to avoid the prime effect (ie, stimulation *in vitro*) to the maximum extent possible. More than 97% of the cells were assessed as being eosinophils based on morphological criteria after May-Grünwald-Giemsa staining. Eosinophil viability was greater than 98% as assessed by trypan blue staining. Eosinophils were suspended in Hank’s balanced salt solution (HBSS) with 0.1% gelatin (HBSS/gel).

### 2.2. *Eosinophil adhesion*

The effect of IL-4, IL-13, and TSLP on the adhesiveness of eosinophils to recombinant human (rh)-ICAM-1-coated plates was evaluated based on the residual eosinophil peroxidase (EPO) activity of adherent eosinophils, as described previously [16-21]. Briefly, 100 μl of eosinophils (1 × 10^4^ cells/well) were stimulated with IL-4 (10 pM–10 nM; R&D Systems, Minneapolis, MN, USA), IL-13 (10 pM–10 nM; R&D Systems), or TSLP (10 pM–10 nM; R&D Systems) in enzyme-linked immuno sorbent assay (ELISA) plates coated with rh-ICAM-1 (10 μg/ml; R&D Systems) at 37°C for 20 minutes. After washing the plate with HBSS 3 times, HBSS/gel (100 μl) was added to each well. After placing standards in empty wells (ie, serially diluted cell suspensions; 1 × 10^2^, 3 × 10^2^, 1 × 10^3^, 3 × 10^3^, and 1 × 10^4^ cells/100 μl), the EPO substrate (1 mM o-phenylenediamine, 1 mM H_2_O_2_, and 0.1% Triton X-100 in Tris buffer, pH 8.0) was added to all wells. The plates were then incubated for 30 minutes and absorbance was measured at 490 nm after stopping the reaction with 4 M H_2_SO_4_. The percentage of eosinophil adhesiveness was determined from mean values calculated from log dose-response curves. Eosinophil viability after incubation was greater than 99%, as assessed by trypan blue staining.

### 2.3. *Eosinophil O*_*2*_^*−*^ *generation*

Eosinophil O_2_^−^ generation was examined using previously described methods based on the superoxide dismutase (SOD)-inhibitable reduction of cytochrome C [16-21]. We first placed 20 μl of SOD (0.2 mg/ml in HBSS/gel) in SOD control wells and then HBSS/gel in all wells of rh-ICAM-1 (10 μg/ml)-coated plates to bring the total volumes to 100 μl. After mixing with cytochrome C, 100 μl of eosinophil suspension (1 × 10^5^ cells/well) was added to all wells. Immediately after adding IL-4 (10 pM–10 nM), IL-13 (10 pM–10 nM), or TSLP (10 pM–10 nM) to the eosinophils, the absorbance of the cell suspensions in the wells was measured at 550 nm, followed by repeated measurements over the next 240 minutes. Each reaction was performed in duplicate and evaluated against the control reaction in wells containing 20 μg/ml of SOD. The results were adjusted for a 1-ml reaction volume, and O_2_^−^ generation was calculated at an extinction coefficient of 21.1 mM^-1^cm^-1^ as nanomoles of cytochrome C reduced per 1.0 × 10^6^ cells/ml minus the SOD control. The maximum value observed over the incubation time was determined. Cell viability, which was assessed by trypan blue staining at the end of each experiment, remained at 97% after 240 minutes of incubation.

### 2.4. *Eosinophil degranulation*

After examining O_2_^−^ generation, the plates were centrifuged immediately, and the recovered cell-free supernatants were measured for EDN, as described previously [16-21]. EDN concentrations were measured by ELISA (Medical and Biological Laboratory Co. Ltd., Nagoya, Japan).

### 2.5. *Statistical analysis*

Values are expressed as means ± SEM. Results were compared using a one-way analysis of variance (ANOVA) followed by the Tukey-Kramer test when differences were significant, or a paired *t* test for analysis of the differences between 2 groups. Values of *P* < 0.05 were considered statistically significant.

## 3. Results

### 3.1. *Effect of IL-4, IL-5, IL-13, and TSLP on eosinophil adhesion*

**Figure 1.**
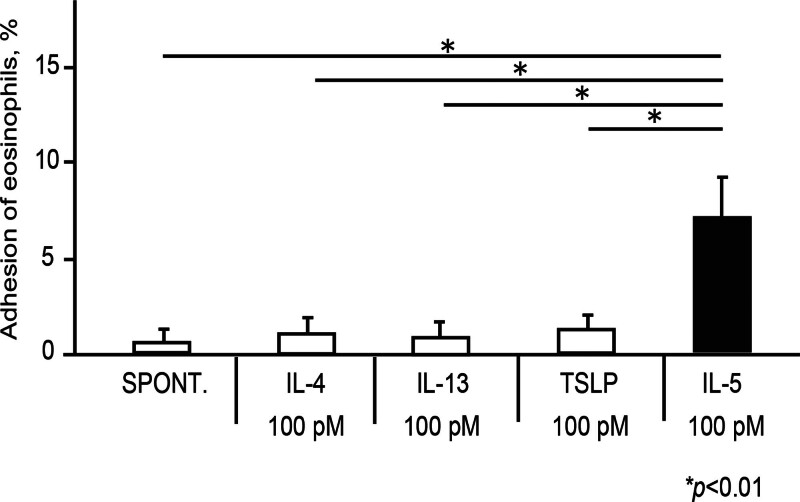
Effect of IL-4, IL-5, IL-13, and TSLP on eosinophil adhesion. Eosinophils (100 μl of 1 × 10^5^ cells/ml in HBSS/gel) obtained from the peripheral blood of healthy donors were incubated with IL-4, IL-13, TSLP, or IL-5 at 100 pM in rh-ICAM-1-coated plates. The adhesiveness of the eosinophils was then assessed by an assay of residual EPO activity. Data are shown as means ± SEM of 5 experiments using cells from different donors. EPO, eosinophil peroxidase; HBSS, Hank’s balanced salt solution; ICAM, intercellular adhesion molecule; IL, interleukin; TSLP, thymic stromal lymphopoietin.

We initially examined the effect of IL-4, IL-5, IL-13, and TSLP on eosinophil adhesion. We stimulated eosinophils with IL-4, IL-13, TSLP, or IL-5 at the same concentration (100 pM) and measured eosinophil adhesiveness to ICAM-1. Although IL-5 at 100 pM directly induced adhesion to ICAM-1, as reported previously [18-20], IL-4, IL-13, or TSLP at 100 pM did not induce eosinophil adhesion (Fig. 1; spontaneous, 0.31 ± 0.32%; IL-4 (100 pM), 0.49 ± 0.51%; IL-13 (100 pM), 0.42 ± 0.47%; TSLP (100 pM), 0.65 ± 0.56%; and IL-5 (100 pM), 7.0 ± 2.7%; *P* < 0.01).

**Figure 2.**
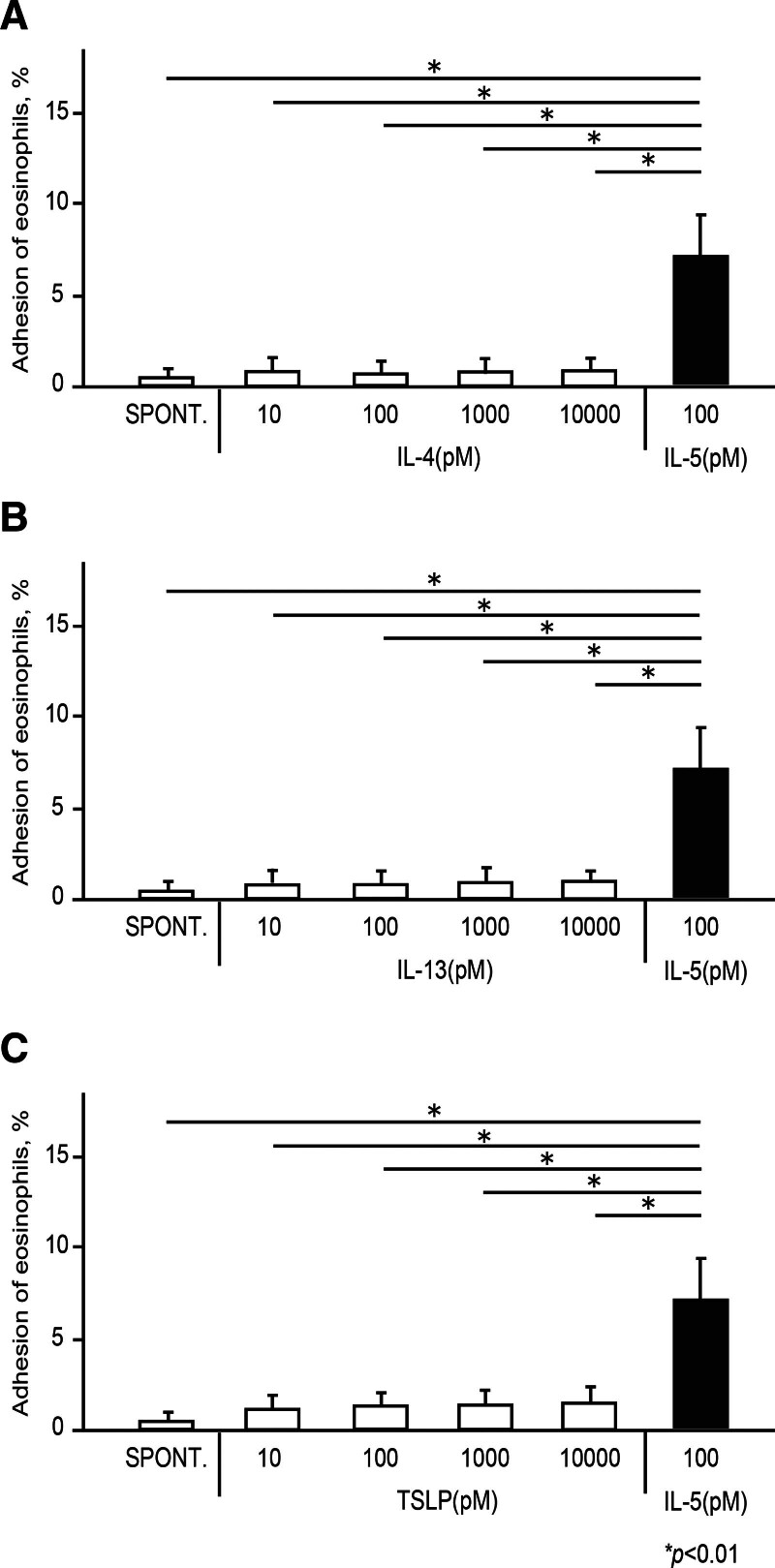
Effect of cytokine concentrations on IL-4, IL-13, and TSLP-induced eosinophil adhesion. Eosinophils (100 μl of 1 × 10^5^ cells/ml in HBSS/gel) obtained from the peripheral blood of healthy donors were incubated with IL-4 (A; 10 pM to 10 nM), IL-13 (B; 10 pM to 10 nM), TSLP (C; 10 pM to 10 nM), or IL-5 (100 pM) in rh-ICAM-1-coated plates. The adhesiveness of the eosinophils was then assessed by assay of residual EPO activity. Data are shown as means ± SEM of 5 experiments using cells from different donors. EPO, eosinophil peroxidase; HBSS, Hank’s balanced salt solution; ICAM, intercellular adhesion molecule; IL, interleukin; TSLP, thymic stromal lymphopoietin.

Next, we examined the effect of higher concentrations of cytokines (IL-4, IL-13, and TSLP) on eosinophil adhesion. Considering the concentrations of these cytokines in serum or airways in bronchial asthma, we selected concentrations of 10 pM to 10 nM [22-29]. At concentrations of 10 pM to 10 nM, IL-4 did not enhance the adhesiveness of eosinophils to ICAM-1 (Fig. 2A; spontaneous, 0.28 ± 0.27%; IL-4 (10 pM), 0.54 ± 0.65%; IL-4 (100 pM), 0.49 ± 0.51%; IL-4 (1 nM), 0.56 ± 0.60%; IL-4 (10 nM), 0.61 ± 0.52%). Similarly, at concentrations of 10 pM to 10 nM, IL-13 did not induce eosinophil adhesion (Fig. 2B; spontaneous, 0.28 ± 0.27%; IL-13 (10 pM), 0.43 ± 0.44%; IL-13 (100 pM), 0.42 ± 0.47%; IL-13 (1 nM), 0.44 ± 0.44%; IL-13 (10 nM), 0.48 ± 0.38%). At concentrations of 10 pM to 10 nM, TSLP also did not increase eosinophil adhesion to ICAM-1 (Fig. 2C; spontaneous, 0.28 ± 0.27%; TSLP (10 pM), 0.60 ± 0.60%; TSLP (100 pM), 0.65 ± 0.57%; TSLP (1 nM), 0.67 ± 0.61%; TSLP (10 nM), 0.70 ± 0.73%). Thus, at concentrations up to 10 nM, IL-4, IL-13, and TSLP were not capable of inducing eosinophil adhesion from the peripheral blood of healthy volunteers.

### 3.2. *Effect of IL-4, IL-5, IL-13, and TSLP on eosinophil O*_*2*_^*−*^ *generation*

**Figure 3.**
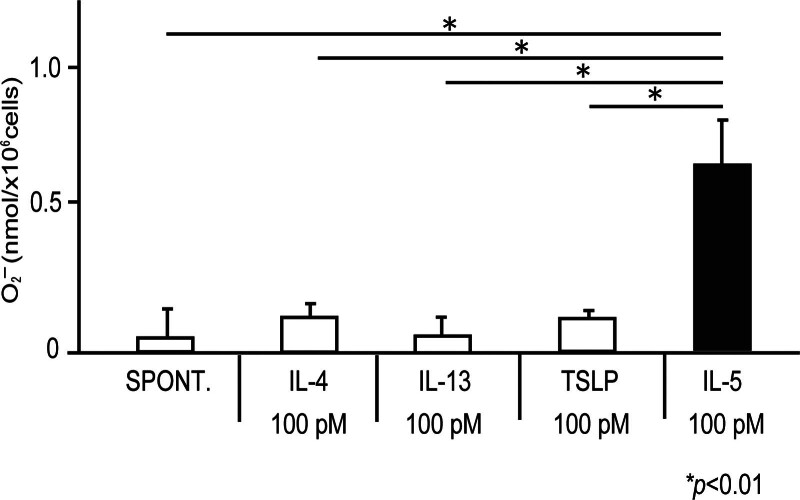
Effect of IL-4, IL-5, IL-13, and TSLP on eosinophil O_2_^−^ generation. Eosinophil cell density was adjusted to 1.25 × 10^6^ cells/ml of HBSS/gel mixed 4:1 with cytochrome C, and the eosinophil suspension was then added to ICAM-1-coated 96-well plates. Immediately after adding IL-4, IL-13, TSLP, or IL-5 at 100 pM, eosinophil O_2_^−^ generation was measured based on the SOD-inhibitable reduction of cytochrome C, followed by measurements repeated over the next 240 min. Maximum values during the incubation time are shown as means ± SEM of 5 experiments using cells from different donors. HBSS, Hank’s balanced salt solution; ICAM, intercellular adhesion molecule; IL, interleukin; SOD, superoxide dismutase; TSLP, thymic stromal lymphopoietin.

Next, we examined whether IL-4, IL-5, IL-13, or TSLP could directly affect eosinophil O_2_^−^ generation. Although IL-5 at 100 pM induced O_2_^−^ generation, as reported previously [18-20], IL-4, IL-13, or TSLP at 100 pM did not induce O_2_^−^ generation of eosinophils from the healthy volunteers (Fig. 3; spontaneous, 0.05 ± 0.12 nmol/10^6^ cells; IL-4 (100 pM), 0.14 ± 0.04 nmol/10^6^ cells; IL-13 (100 pM), 0.06 ± 0.07 nmol/10^6^ cells; TSLP (100 pM), 0.11 ± 0.01 nmol/10^6^ cells; IL-5 (100 pM), 0.56 ± 0.17 nmol/10^6^ cells; *P* < 0.01). We then examined the effect of IL-4, IL-13, or TSLP concentrations on cytokine-induced eosinophil O_2_^−^ generation. At concentrations of 10 pM to 10 nM, IL-4, IL-13, or TSLP did not induce O_2_^−^ generation in eosinophils; similar to data obtained for eosinophil adhesion (data not shown).

### 3.3. *Effect of IL-4, IL-5, IL-13, and TSLP on EDN release*

**Figure 4.**
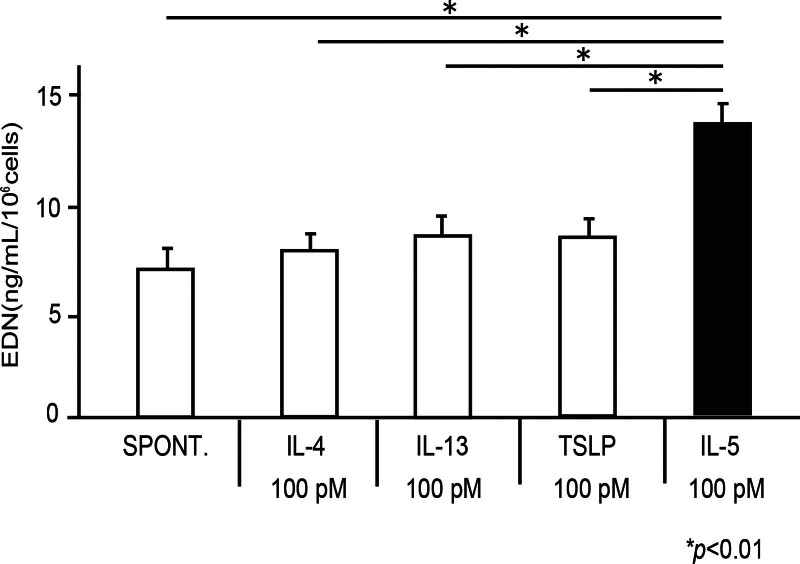
Effect of IL-4, IL-5, IL-13, and TSLP on eosinophil EDN release. Eosinophils (1 × 10^6^ cells/ml) in 96-well plates were incubated with IL-4, IL-13, TSLP, or IL-5 at 100 pM for 240 min. The concentration of EDN in cell-free supernatants was then quantified using ELISA. Data are shown as means ± SEM of 5 experiments using cells from different donors. EDN, eosinophil-derived neurotoxin; ELISA, enzyme-linked immuno sorbent assay; ICAM, intercellular adhesion molecule; IL, interleukin; TSLP, thymic stromal lymphopoietin.

Finally, we examined the effect of IL-4, IL-5, IL-13, and TSLP on the degranulation of eosinophils. Although IL-5 induced EDN release at 100 pM, as reported previously [18-20], IL-4, IL-13, or TSLP did not induce EDN release at 100 pM in eosinophils from healthy volunteers (Fig. 4; spontaneous, 7.19 ± 0.72 ng/ml/10^6^ cells; IL-4 (100 pM), 8.11 ± 0.54 ng/ml/10^6^ cells; IL-13 (100 pM), 8.84 ± 0.53 ng/ml/10^6^ cells; TSLP (100 pM), 8.74 ± 0.55 ng/ml/10^6^ cells; IL-5 (100 pM), 13.3 ± 0.95 ng/ml/10^6^ cells; *P* < 0.01). Next, we examined the effect of IL-4, IL-13, or TSLP concentrations on cytokine-induced EDN release. At concentrations of 10 pM to 10 nM, IL-4, IL-13, or TSLP did not induce EDN release (data not shown).

## 4. Discussion

In this study, we found that IL-4, IL-13, and TSLP at concentrations up to 10 nM did not induce eosinophil adhesiveness to ICAM-1, O_2_^−^ generation, or EDN release in eosinophils obtained from the peripheral blood of healthy individuals. On the other hand, at 100 pM, IL-5 induced eosinophil adhesiveness, O_2_^−^ generation, and EDN release, as reported previously [18-20]. These findings suggest that anti-IL-4Rα Ab and anti-TSLP Ab suppress eosinophilic inflammation in severe asthma, most likely through indirect mechanisms.

In this study, we selected cytokine concentrations ranging from 10 pM to 10 nM, considering that these are the concentrations of these cytokines in serum and in the airways in bronchial asthma [22-29]. To elucidate the mechanisms of action of biologics targeting type 2 cytokines, we examined the direct effect of cytokines on eosinophils at these concentrations. The concentrations of cytokines such as IL-4, IL-5, IL-13, and TSLP in serum, sputum, and bronchoalveolar lavage fluid (BALF) in asthma have been reported to range from 1 to 100 pg/ml (approximately 0.7–70 pM) [22-27]. Cytokine concentrations are higher in children with asthma than in those in adults and have been reported to range from 100 to 1000 pg/ml (approximately 70–700 pM) [28, 29]. For example, Couillard et al. [22] reported that the mean serum concentrations of IL-4, IL-5, IL-13, and TSLP in eosinophilic asthma are 0.1, 0.8, 3.3, and 1.8 pg/ml, respectively. Ko et al. [23] reported that the serum concentrations of IL-5, IL-13, and TSLP in asthma prone to exacerbation are 2.1, 63.8, and 16.3 pg/ml, respectively. Berraïes et al. [28] reported that the serum concentration of TSLP in children with asthma is 106 ± 51 pg/ml. As for sputum, Couillard et al. [22] reported that sputum concentrations of IL-4, IL-5, IL-13, and TSLP in eosinophilic asthma are 0.3, 4.7, 7.6, and 7.7 pg/ml, respectively [22]. Manise et al. [24] reported that sputum concentrations of IL-4, IL-5, and IL-13 in eosinophilic asthma are 0, 6, and 11 pg/ml. Berraïes et al. [28] reported that the TSLP concentration in sputum from children with asthma is 240 ± 131 pg/ml. Vizmanos-Lamotte et al. [29] reported that sputum IL-4 concentration in children with atopic asthma is approximately 1000 pg/ml, although IL-4 concentration was assessed by flow cytometry. As for BALF, Li et al. [25] reported that the IL-4, IL-5, IL-13, and TSLP concentrations of BALF in severe asthma cases are approximately 1, 1, 3, and 30 pg/ml, respectively. Liu et al. [26] reported that the TSLP concentration of BALF in asthma was approximately 50 pg/ml, although that in disease controls was approximately 20 pg/ml. Collectively, in this study, we examined the direct effect of cytokines on eosinophils at clinical or slightly higher concentrations.

At concentrations of up to 10 nM, neither IL-4 nor IL-13 induced eosinophil adhesion, O_2_^−^ production, or degranulation in eosinophils obtained from healthy individuals. Few studies have examined the direct effect of IL-4 or IL-13 on eosinophil function [6, 7, 30-32]. For example, Dubois et al. [30] reported that although IL-4 did not induce the chemotaxis of eosinophils from healthy individuals, even at a concentration of 10 nM, IL-4 induced eosinophil chemotaxis in patients with atopic dermatitis at concentrations greater than 1 pM. As for eosinophils from healthy individuals, IL-4 could induce chemotactic responses at concentrations greater than 10 pM, following pretreatment with IL-5 or granulocyte-macrophage colony-stimulating factor (GM-CSF) (10 pM) [30]. In a subsequent study, these authors also reported that human eosinophils express a functional IL-4 receptor [6]. Although IL-4 alone did not induce eosinophil chemotactic responses, at a concentration of 1 nM, IL-4 increased regulated on activation, normal T cell expressed and secreted-induced, but not platelet-activating factor-induced, eosinophil chemotaxis, and phosphatidylinositol-3 kinase activity in eosinophils [6]. As for the effect of IL-4R on eosinophils, Spencer et al. [7] reported that the IL-4R α-chain and γc are mainly expressed inside eosinophils and not at the surface, suggesting that IL-4 plays a limited role in eosinophil activation. Recently, Miyata et al. [31] reported that IL-4 at 10 ng/ml (approximately 0.8 nM) induced the expression of gamma-glutamyltransferase 5 mRNA, which may be important for LTD4 synthesis, in eosinophils; however, the effect of IL-4 on the actual synthesis of cysteinyl leukotrienes by eosinophils was not described in their study. As for IL-13, Horie et al. [32] investigated the effects of IL-13 on eosinophil chemotaxis and survival. They found that at concentrations more than 3 ng/ml (approximately 0.25 nM), IL-13 increased eosinophil survival and that at concentrations greater than 50 ng/ml (approximately 4 nM), IL-13 induced eosinophil chemotaxis [32]. The increase in eosinophil survival by IL-13 was suppressed by treatment with mAbs against IL-3 or GM-CSF, suggesting that IL-3 and GM-CSF produced from IL-13-stimulated eosinophils increases their survival in an autocrine-type manner [32]. However, the effect of IL-13 on eosinophil survival was considerably lower than that of IL-5; for example, the concentration of IL-13 that was required to achieve 50% survival of eosinophils was more than 10,000-fold that of IL-5 [32]. In their study, even at a concentration of 250 ng/ml (approximately 17 nM), IL-4 neither increased eosinophil survival nor did it induce chemotaxis [32]. Similarly, even at a concentration of 250 ng/ml, IL-13 did not release EDN [32]. Taken together, the direct effects of IL-4 and/or IL-13 on the effector functions of eosinophils are generally negligible, and are probably, at least in part, dependent on experimental conditions such as eosinophil purification methods and incubation time. Even if eosinophils are activated by IL-4 or IL-13, the concentrations of these cytokines that are required to activate eosinophils are considerably higher than that of IL-5, suggesting that their impact on eosinophil functioning is markedly less than that of IL-5. Moreover, several studies have suggested that IL-4 and IL-13 may induce eosinophil-effector functions such as chemotaxis under specific conditions, for example, if eosinophils are obtained from patients with atopic dermatitis or after pretreatment with eosinophil-activating cytokine/chemokines such as IL-5, GM-CSF, or regulated on activation, normal T cell expressed and secreted, which were not examined in this study.

It is well established that IL-4 and IL-13 can affect the development of eosinophilic airway inflammation through indirect mechanisms [5, 33, 34]. For example, during the process of eosinophil accumulation in the airway, peripheral blood eosinophils must adhere to vascular endothelial cells and then transmigrate across endothelial cells to the lung. Vascular cell adhesion molecule (VCAM)-1 on vascular endothelial cells, which is induced by IL-4 or IL-13, plays an important role in this selective eosinophil adhesiveness [35-39]. After eosinophil adhesion, C-C chemokine receptor type 3 (CCR-3) ligands including eotaxin, which are also induced by IL-4 or IL-13 [40, 41], increase eosinophil transmigration across VCAM-1-expressing vascular endothelial cells [42, 43]. Furthermore, periostin, an extracellular matrix protein that is expressed in the airways of bronchial asthma [44] and also induced by IL-4 or IL-13, can activate eosinophils *in vitro* [18]. Therefore, IL-4 or IL-13-induced proteins, such as VCAM-1, CCR-3 ligands, and periostin, likely contribute to the development of eosinophilic airway inflammation and activation through indirect mechanisms.

TSLP is an epithelial cell-related cytokine that activates several immune cells, such as ILC2, DCs, and basophils that express the TSLPR [9]. Innate immune responses also contribute to the development of eosinophilic inflammation in the airway, mainly through epithelial cell-related cytokines including IL-33, TSLP, IL-25, and ILC2 [8]. TSLP can enhance the survival of ILC2, which can produce IL-5 and IL-13 and thus induce eosinophilic inflammation. In severe asthma, TSLP plays a role in the development of corticosteroid resistance in airway inflammation through B-cell lymphoma-extra large expression by ILC2s [26, 45]. As for adaptive immune responses, TSLP contributes to DC activation and Th2 differentiation. TSLP increases the expression of OX40L, an important inducer of Th2, in DCs and thus promotes the differentiation of naive T cells into Th2 cells [46]. DCs themselves produce TSLP, suggesting that autocrine TSLP may contribute to the induction of Th2 inflammation [47]. Moreover, TSLP induces basophil differentiation as well as basophil activation in an IL-3-independent manner. Salter et al. [48] reported that TSLPR expression of basophils in the asthmatic airways is upregulated after allergen challenge. In addition, TSLP induces basophil activation marker CD203c expression, type 2 cytokine production, histamine release, and eotaxin-mediated migration [48]. TSLP also increases the expression of IL-17RB and ST2, the receptors for IL-25 and IL-33 respectively, in basophils, suggesting that TSLP can increase IL-25- or IL-33-mediated basophil activation [49]. Therefore, various types of cells, including ILC2, DCs, and basophils, play roles in the development of TSLP-induced eosinophil airway inflammation. However, the most important mechanisms for the development of eosinophilic airway inflammation by TSLP have not been fully clarified. In a study involving the targeted deletion of TSLPR in mice, Kabata et al. [50] reported that ILC2, not basophils, are essential for the development of TSLP-induced innate immune-mediated eosinophilic inflammation [50]. They also demonstrated that DCs and CD4+ T cells, but not basophils or ILC2s, were required for TSLP-induced adaptive immune-mediated eosinophilic inflammation [50]. In clinical studies, anti-TSLP Ab treatment was shown to suppress asthma exacerbation in patients with severe asthma, [51] inhibit eosinophilic inflammation in the airway, and to decrease the concentrations of IL-5 and IL-13 in serum [15]. Based on the findings of targeted deletion of the TSLPR in mice, it is assumed that ILC2 and DCs/CD4+ T cells play an important role in the pathogenesis of TSLP-induced innate and adaptive immune-mediated eosinophilic airway inflammation in humans, which should be examined in the future.

In this study, at concentrations of up to 10 nM, TSLP did not induce eosinophil adhesion, O_2_^−^ generation, or eosinophil degranulation in eosinophils obtained from healthy individuals. Several studies have suggested that TSLPR is also expressed on eosinophils [10] and that TSLP directly activates eosinophils *in vitro* [10-12]. However, these studies suggested that concentrations higher than clinical concentrations are needed to activate eosinophils *in vitro* [10-12]. For example, Wong et al. [10] reported that at 50 ng/ml (approximately 3.3 nM), TSLP-induced eosinophil adhesion to fibronectin and enhanced CD18 and ICAM-1 expression, and that at 20 ng/ml (approximately 1.3 nM), TSLP suppressed apoptosis and increased eosinophil survival. They also reported that at 50 ng/ml, TSLP induced the production of cytokines or chemokines, including IL-6, CXCL8, CXCL1, and CCL2, from eosinophils; however, TSLP did not induce eosinophil cationic protein release from eosinophils [10]. Cook et al. [11] reported that IL-3 and tumor necrosis factor-α increased the expression of TSLP receptor on eosinophils and that TSLP-induced EDN release and increased survival at 1 μg/ml (approximately 660 nM) [11]. They also reported that pretreatment of eosinophils with IL-3 and tumor necrosis factor-α increased TSLP-induced eosinophil degranulation [11]. Furthermore, Morshed et al. [12] reported that TSLP induced the eosinophil extracellular trap formation [12]. At 20 ng/ml, TSLP-induced eosinophil extracellular traps through an adhesion-dependent and reactive oxygen species production-dependent manner. Whether TSLP activates the eosinophils of patients with atopic dermatitis or allergic patients more than it activates those of healthy individuals has not been fully elucidated. Taken together, TSLP at clinical concentrations in serum or sputum did not induce eosinophil effector functions, but concentrations higher than clinical concentrations may activate eosinophils.

In this study, we measured EDN as a marker of eosinophil degranulation. Although we have not compared the EDN with other eosinophil granule proteins, we have measured EDN, considering the advantages of its measurement. For example, EDN is superior to eosinophil cationic protein in test reproducibility [52]. EDN is more easily recovered from measuring instruments and cell surfaces because of its weaker electrical charge [53]. EDN is also known to be released from eosinophils at a greater efficiency than eosinophil cationic protein [54].

A limitation of this study was that we only used eosinophils from the peripheral blood of healthy volunteers. We did a preliminary study using eosinophils from patients with asthma and found IL-4, IL-13, or TSLP at concentrations up to 10 nM did not induce eosinophil adhesion, O_2_^−^ generation, or eosinophil degranulation (data not shown). However, we have not examined the effect of these cytokines on the function of eosinophils from severe asthmatics. There is a possibility that IL-4R or TSLPR is induced or increased in eosinophils from patients with severe asthma or atopic dermatitis, and these eosinophils may respond to IL-4, IL-13, or TSLP at concentrations up to 10 nM. In fact, some articles suggest the upregulation of IL-4R or TSLPR in the immune cells of patients with asthma. For example, Baba et al. [55] reported that IL-4R expression of ILC2 is higher in asthmatics than in healthy individual. Furthermore, Paplińska-Goryca et al. [56] reported that TSLPR expression of macrophages is higher in asthmatics than in healthy volunteers. Furthermore, eosinophils at the site of inflammation, such as airways, may also respond to cytokines at lower concentrations. Therefore, it is possible that expression of IL-4R or TSLPR of eosinophils is induced or increased in asthmatics, especially in severe asthmatics, and IL-4/IL-13 or TSLP at clinical concentrations directly activates eosinophils, especially airway eosinophils, from asthmatics. Another limitation is that we did not examine the additional effect of IL-4, IL-13, or TSLP on other types of cytokine/chemokine-induced eosinophil activation, as described above.

In conclusion, at concentrations of up to 10 nM, IL-4, IL-13, and TSLP did not activate eosinophils, while at a concentration of 100 pM, IL-5 intensely activated eosinophils. By themselves, type 2 cytokines other than IL-5 cannot directly affect eosinophil functions at either clinical or slightly higher concentrations. These findings suggest that eosinophils play little, or no, direct role in the effects of anti-IL-4Rα or anti-TSLP Ab on severe asthma.

## Conflicts of interest

The authors have no financial conflicts of interest.

## Author contributions

YU carried out the experiments, analyzed the data, and drafted the manuscript. KN participated in direction of the study, analyzed the data, and wrote the manuscript. KK, HI, SM, TN, and TK carried out the eosinophil experiments. TS and TI participated in the data analyses. MN participated in direction of the study and edited the manuscript. All of the authors have read and approved the final manuscript.
